# Calcium isotope ratios in blood and urine: A new biomarker for the diagnosis of osteoporosis

**DOI:** 10.1016/j.bonr.2019.100200

**Published:** 2019-03-16

**Authors:** A. Eisenhauer, M. Müller, A. Heuser, A. Kolevica, C.-C. Glüer, M. Both, C. Laue, U.v. Hehn, S. Kloth, R. Shroff, J. Schrezenmeir

**Affiliations:** a GEOMAR Helmholtz Centre for Ocean Research Kiel, 24148 Kiel, Wischhofstr.1-3, Germany; b University Medical Center Schleswig-Holstein (UKSH), Arnold-Heller-Str. 3, 24105 Kiel, Germany; c Sektion Biomedizinische Bildgebung, Klinik für Radiologie und Neuroradiologie, Am Botanischen Garten 14, 24118 Kiel, Germany; d Klinik für Neuroradiologie und Radiologie, (UKSH), Arnold-Heller-Str. 3, 24105 Kiel, Germany; e Clinical Research Center Kiel GmbH, Schauenburgerstraße 116, 24118 Kiel, Germany; f Medistat, GmbH, Kieler Straße 15, 24119 Kronshagen, Germany; g OSTEOLABS GmbH, c/o GEOMAR Helmholtz Centre for Ocean Research Kiel, 24148 Kiel, Wischhofstr.1-3, Germany; h Great Ormond Street Hospital for Children NHS Foundation Trust, London WC1N 3JH, United Kingdom of Great Britain and Northern Ireland

**Keywords:** Osteoporosis, Bone mineral density (BMD), Dual energy x-ray absorptiometry (DXA), Calcium isotopes, Bone biomarkers, Mass-spectrometry

## Abstract

We assessed the potential of Calcium (Ca) isotope fractionation measurements in blood (δ^44/42^Ca_Blood_) and urine (δ^44/42^Ca_Urine_) as a new biomarker for the diagnosis of osteoporosis. One hundred post-menopausal women aged 50 to 75 years underwent dual-energy X-ray absorptiometry (DXA), the gold standard for determination of bone mineral density. After exclusion of women with kidney failure and vitamin D deficiency (<25 nmol/l) 80 women remained in the study. Of these women 14 fulfilled the standard diagnostic criteria for osteoporosis based on DXA. Both the δ^44/42^Ca_Blood_ (*p* < 0.001) and δ^44/42^Ca_Urine_ (*p* = 0.004) values were significantly different in women with osteoporosis (δ^44/42^Ca_Blood_: −0.99 ± 0.10‰, δ ^44/42^Ca_Urine_: +0.10 ± 0.21‰, (Mean ± one standard deviation (SD), *n* = 14)) from those without osteoporosis (δ^44/42^Ca_Blood_: −0.84 ± 0.14‰, δ^44/42^Ca_Urine_: +0.35 ± 0.33‰, (SD), *n* = 66). This corresponded to the average Ca concentrations in morning spot urine samples ([Ca]_Urine_) which were higher (*p* = 0.041) in those women suffering from osteoporosis ([Ca]_Urine-Osteoporosis_: 2.58 ± 1.26 mmol/l, (SD), *n* = 14) than in the control group ([Ca]_Urine_-_Control_: 1.96 ± 1.39 mmol/l, (SD), *n* = 66). However, blood Ca concentrations ([Ca]_Blood_) were statistically indistinguishable between groups ([Ca]_Blood_, control: 2.39 ± 0.10 mmol/l (SD), *n* = 66); osteoporosis group: 2.43 ± 0.10 mmol/l (SD, *n* = 14) and were also not correlated to their corresponding Ca isotope compositions. The δ^44/42^Ca_Blood_ and δ^44/42^Ca_Urine_ values correlated significantly (*p* = 0.004 to *p* = 0.031) with their corresponding DXA data indicating that both Ca isotope ratios are biomarkers for osteoporosis. Furthermore, Ca isotope ratios were significantly correlated to other clinical parameters ([Ca]_Urine_, ([Ca]_Urine/_Creatinine)) and biomarkers (CRP, CTX/P1NP) associated with bone mineralization and demineralization. From regression analysis it can be shown that the δ^44/42^Ca_Blood_ values are the best biomarker for osteoporosis and that no other clinical parameters need to be taken into account in order to improve diagnosis. Cut-off values for discrimination of subjects suffering from osteoporosis were − 0.85‰ and 0.16‰ for δ^44/42^Ca_Blood_ and δ^44/42^Ca_Urine_, respectively. Corresponding sensitivities were 100% for δ^44/42^Ca_Blood_ and ~79% for δ^44/42^Ca_Urine_. Apparent specificities were ~55% for δ^44/42^Ca_Blood_ and ~71%. The apparent discrepancy in the number of diagnosed cases is reconciled by the different methodological approaches to diagnose osteoporosis. DXA reflects the bone mass density (BMD) of selected bones only (femur and spine) whereas the Ca isotope biomarker reflects bone Ca loss of the whole skeleton. In addition, the close correlation between Ca isotopes and biomarkers of bone demineralization suggest that early changes in bone demineralization are detected by Ca isotope values, long before radiological changes in BMD can manifest on DXA. Further studies are required to independently confirm that Ca isotope measurement provide a sensitive, non-invasive and radiation-free method for the diagnosis of osteoporosis.

## 1 Introduction

Calcium is an essential mineral in the body, controlling the formation and maintenance of bones and teeth (Boskey, 2008), muscle contraction, neural signal transmission, cell apoptosis and the coagulation of blood. Calcium homeostasis is carefully regulated through hormonal feedback loops in order to keep blood Ca levels within a narrow therapeutic range (Gussone, 2016). Essentially, three organs control the Ca homeostasis: the gastrointestinal tract (GI), where Ca is absorbed from the diet, the skeleton which is the main Ca reservoir, and the kidneys where Ca is reabsorbed from the urine back into the blood and controlled amounts are excreted. Physiological ageing processes as well as intestinal, bone, kidney and endocrine disorders interfere with Ca homeostasis. Osteoporosis, osteomalacia and rickets as well as renal osteodystrophy are examples of bone disorders linked to an impaired Ca metabolism. Osteoporosis is the most widely prevalent condition, mainly affecting postmenopausal women. It is a systemic disorder characterized by low BMD, increased fragility and predisposition to fractures (Consensus development conference: diagnosis, prophylaxis, and treatment of osteoporosis, 1993). In clinical practice osteoporosis is diagnosed when BMD is reduced on DXA, after exclusion of other causes (Consensus development conference: diagnosis, prophylaxis, and treatment of osteoporosis, 1993; Kanis, and WHO, Scientific, Group, 2007).

Changes in BMD are associated with a net loss of Ca from the bone. This has been investigated by tracer experiments using stable (non-radioactive) Ca isotopes that are naturally present in the environment such as ^42^Ca and ^44^Ca (Abrams, 1994; Abrams et al., 1994) as well as with radioactive Ca isotopes of different half-lives (^41^Ca, ^45^Ca or ^47^Ca) (Abrams, 1994; Abrams et al., 1994; Abrams, 1999; Beck et al., 2003; Feeman et al., 1996; Heaney and Nordin, 2002; Stürup et al., 1997). Tracer studies have been carried out by the simultaneous administration of tracer enriched Ca orally and intravenously. However these experiments are expensive, require a ‘metabolic cage’ like setting, and, in case of radioactive isotopes, are associated with harmful radiation. In contrast, studies using stable Ca isotopes naturally present in our diet, are much cheaper and avoid exposure to radioactivity (Skulan et al., 2007; Skulan and DePaolo, 1999a; Heuser and Eisenhauer, 2010).

The application of natural Ca isotopes follows the principle of kinetic isotope fractionation that separates stable isotopes from each other as a function of their mass during unidirectional biochemical processes (Eisenhauer et al., 2009; Bullen and Eisenhauer, 2009). As a consequence of kinetic isotope fractionation, in a chain of chemical reactions the lighter isotope always becomes enriched in the product. Thus, at a trophic level along the food chain, lighter isotopes become enriched from plants to humans. This is expressed as decreasing ^44^Ca/^42^Ca ratios (reported as δ^44/42^Ca) from vegetables via meat to humans (Chu et al., 2005), with human mother's milk being the most enriched reservoir for light Ca isotopes. Similarly, in the human body δ^44/42^Ca isotope ratios decrease as a function of biochemical processes whereby Ca from the diet goes via the blood into the skeleton (Heuser et al., 2016). This means that major Ca compartments in our body, such as blood and soft tissues, bone, urine and feces, are well characterized with a Ca isotope “fingerprint” and can be used for diagnostic purposes in order to identify distinct Ca related disorders. Different laboratories have quantitatively confirmed that the Ca isotope fractionation, and hence the isotope difference between blood and bone (Δ^44/42^Ca_Bone-Blood_ = δ^44/42^Ca_Bone_ - δ^44/42^Ca_Blood_) is fairly constant in the order of about −0.3‰, in different vertebrate species including chickens, horses (Skulan et al., 1997; Skulan and DePaolo, 1999b), “Göttingen” mini-pigs (Heuser and Eisenhauer, 2010) and humans (Morgan et al., 2012a), and this value is independent of the absolute Ca isotope values in blood and bone.

Based on observational data in animal and human studies (Heuser and Eisenhauer, 2010; Skulan et al., 1997; Skulan and DePaolo, 1999b; Morgan et al., 2012a) Ca isotopes in blood and urine could theoretically be used to study disturbances in bone mineral balance such as in osteoporosis. This is based on the principal that the blood and bone Ca isotope compositions are in isotopic equilibrium only differing by a constant value (Δ^44/42^Ca_Bone-Blood_ = δ^44/42^Ca_Bone_ - δ^44/42^Ca_Blood_ = −0.3‰) (Heuser and Eisenhauer, 2010; Skulan et al., 1997; Skulan and DePaolo, 1999b; Morgan et al., 2012a). During equilibrium between bone material absorption and resorption the blood Ca isotope value is not changing. Any short term disturbances of the isotope equilibrium between absorption and resorption (like an increase in dietary Ca intake) is re-equilibrated again within a half-life of about an hour due to low blood Ca concentrations and the high Ca exchange rates between bone and blood. However, when bone resorption is persistently greater than bone absorption, as seen in osteoporosis, the blood Ca isotope composition becomes lower. When the disequilibrium between Ca absorption and resorption continues for days to years, the bone Ca isotope composition successively decreases as a function of time towards lower and lower values also reflected in the blood Ca isotope composition by lower and lower values. The ongoing loss of Ca then reflects a decrease of the BMD detectable by DXA at a certain time. Hence, a relationship between Ca isotope values in blood and urine to bone DXA values is predicted and may eventually be applied to support the diagnosis of osteoporosis. These theoretical inferences have not been studied in humans.

We undertook this study to determine the feasibility and diagnostic accuracy of Ca isotope fractionation measured in blood and urine for the diagnosis of osteoporosis in post-menopausal women, comparing against their corresponding DXA values as the gold standard. The positive and negative predictive value of Ca isotope fractionation were determined and the association of δ^44/42^Ca_Urine_ and δ^44/42^Ca_Blood_ with biomarkers of bone resorption, (CTX; C-terminal telopeptide), bone formation (P1NP; N-terminal propeptide of type 1 procollagen) and inflammation (CRP; C-reactive protein) ((WHO, 1994; Felsenberg et al., 1998), were studied.

## 2 Methods

The aim of this study was to investigate whether the natural Ca isotope ratios (δ^44/42^Ca) in urine (δ^44/42^Ca_Urine_) and blood (δ^44/42^Ca_Blood_) can be used to diagnose osteoporosis in postmenopausal women, and the diagnostic accuracy of the Ca isotope fractionation technique compared to the gold standard DXA scans and bone biomarkers.

### 2.1 Study design

The study was approved and registered (NCT02967978) by an independent ethics committee (The Ethical Committee of the Medical Council of Schleswig-Holstein, Bad Segeberg, Germany) and conducted according to the principles stated in the Declaration of Helsinki, the national and international guidelines of the “Dachverband für Osteoporose” (DVO) 2014 and the International Society of Clinical Densitometry (ISCD) 2015 (DVO, n.d.; ISCD, n.d.), respectively.

Post-menopausal women aged 50–75 years who had at least one risk factor for osteoporosis by age 60 years or at least 2 risk factors by age 50 years, were invited to participate in the study. Women with a known fracture within the previous 3 months, those with renal failure, cancer, and hyperparathyroidism or on sex hormone treatment were excluded. Women with Vitamin D deficiency (defined as 25 Hydroxy Vitamin D level <25 nmol/l, as per the DVO definition) were excluded. In general, the definition of Vitamin D deficiency lightly vary in different national and international guidelines as defined by IOF (*International Osteoporosis Foundation*) and IOM (*Institute of Medicine*). According to IOF (IOM), 25OHD levels of <20 ng/ml (<12 ng/ml) is termed as deficiency, 20–30 ng/ml (12–20 ng/ml) as insufficiency, and levels >30 ng/ml (>20 ng/ml) as sufficiency. We have used the German national DVO recommendation which is in fact a mean value of IOM and IOF (<25 ng).

One hundred consecutive women fulfilling the above criteria were screened at the Clinical Research Centre (CRC), Kiel. All patients underwent a DXA scan of the lumbar spine and proximal femur. Women were divided into those with osteoporosis and those without osteoporosis according to the world health organization (WHO) definition (WHO, 1994): osteoporosis was considered present if BMD as assessed by DXA densitometry of the lumbar spine and/or proximal femur (total area or femoral neck) had a T-Score ≤ −2.5, and after excluding other disorders associated with a reduction in bone mineral content and osteomalacia. We based the recruitment on the following assumptions: according to the European Prospective Osteoporosis Study (EPOS) study (Felsenberg et al., 1998) the prevalence of osteoporosis in German postmenopausal women aged 50 to 60 years is about 15% and about 45% at the age of >70 years. In women with risk factors such as hip fracture in father and/or mother, smoking, lactose intolerance, vegan diet, underweight (BMI < 20), diabetes, or long-standing use of loop diuretics, glucocorticoids, aromatase inhibitors and proton pump inhibitors, a higher prevalence is expected. Thus, by selecting women with at least one risk factor for osteoporosis, a higher proportion than in the general population were expected to meet the criteria for osteoporosis. Based on these assumptions, we expected to have at least 40% subjects suffering from osteoporosis. On screening 100 women, 20 women were found to have low vitamin D levels, renal failure or other risk factors requiring exclusion. Eighty women (*n* = 40) with risk factors for osteoporosis and *n* = 40 without risk factors for osteoporosis (age-matched control group) were finally included in the study.

#### 2.1.1 Study investigations

At the first screening visit demographic details, past medical history, including risk factors for osteoporosis, and medication history were determined and suitable subjects consented and recruited. Weight and height measurements were performed and dietary intake was assessed using food-frequency-questionnaires (FFQ). At the second visit women were asked to bring in a single morning urine and fecal sample of that day, and blood sampling was performed. DXA of lumbar vertebrae and hip was performed within 4 weeks after visit 2. An optional visit 3 served for informing the subjects about their findings.

### 2.2 Dual X-ray absorptiometry (DXA)

DXA of the lumbar spine and hip were performed on a Hologic QDR 4500 following manufacturer's instructions and standard protocol. All images were analyzed by observers blinded to the subjects' clinical status. According to the WHO definition, osteoporosis is present when the bone density measurement is 2.5 SD below the average of 20–29 year-old women (peak bone mass), also expressed as a T-score value ≤ −2.5. A T-score value of ≤ −2.5 indicates significantly reduced bone density and an increased risk of bone fractures. T-scores between −1 and −2.5 SD are defined as reduced bone density or osteopenia. A T score value ≥ −1 is considered to reflect healthy bones. In this study, the lowest T-score obtained from either vertebral spine or hip was used for the diagnosis of osteoporosis. Subjects in whom the DXA quality was poor due to scoliosis, degenerative disease or other issues were excluded from further analysis.

### 2.3 Chemical sample preparation and mass-spectrometer measurement

#### 2.3.1 Sample digestion and purification

Samples were chemically treated in batches to remove all organic material before measurement in the mass spectrometer. Each batch consisting out of 20 samples (acidified prior to chemical treatment), one sample replicate, one procedural blank, one of each reference material (NIST SRM 915a, NIST SRM 1486, IAPSO Standard Seawater) and one of the in-house standards (urine AK1 or blood SERA-1). All liquid samples were homogenized by shaking prior to chemical preparation. About 1180 μl of acidified urine, 450 μl serum and about 100 to 500 mg feces were used for chemical digestion. About 3 ml of 14 mol/l HNO_3_ and 1 ml of H_2_O_2_ (30%) were added to urine and feces and 3 ml of 14 mol/l HNO_3_ plus 100 μl HClO_4_ were added to the serum samples. Blank and reference materials were always treated the same way. The closed beakers were placed on a hot plate at 120 °C for 12 h then opened and evaporated afterwards. The residues were treated with nitric acid and hydrogen peroxide, heated to 120 °C and evaporated, with the procedure repeated multiple times until the solution was colorless. Next, Ca, Magnesium (Mg) and Strontium (Sr) concentrations of the solutions were measured on a Q-ICP-MS Agilent 7500cx. An aliquot of the sample solution containing 50 μg of Ca was chemically purified using an automatic purification system (prepFAST MC, ESI, USA). Calcium yields were in the order of or better than 95% and the Ca/Sr in the processed solution were >10000. The Ca fraction collected from the original solutions was dried down at 120 °C and then re-dissolved in 1 ml of 14 mol/l HNO_3_ and 0.5 ml H_2_O_2_ (30%). The solution was then heated up with closed lids for 12 h at 120 °C and dried down again. Finally the residues were re-dissolved in 0.2 mol/l nitric acid to yield a final Ca concentration of about 5 μg/ml.

#### 2.3.2 Mass spectrometry and data evaluation

Calcium isotope measurements were performed on a MC-ICP-MS (Neptune plus, Thermo Fisher Scientific, Bremen, Germany) at the mass spectrometer facilities of the GEOMAR Helmholtz Centre for Ocean Research Kiel, Germany. The mass spectrometer was equipped with nine Faraday cups of which eight are moveable. The mass spectrometer was set up to measure masses 42, 43, 43.5 and 44 simultaneously. In order to suppress interfering Ca- and Ar-hydrides (e.g. ^40^Ar^1^H_2_ on ^42^Ca) an APEX IR (ESI, Omaha, Nebraska, USA) sample introduction system was used. All measurements were performed in medium resolution (MR, m/Δm~4000) on the interference-free plateau of the low mass side of the peaks. This was achieved by choosing an appropriate center cup mass of 43.687 ± 0.001 amu and verified on a daily basis (cf. (Wieser et al., 2004)). Instrumental fractionation (mass bias) was corrected by applying the standard-sample-bracketing approach. The measurement of a sample was bracketed by measurements of a ~5 μg/ml Ca solution prepared from a 10,000 μg/g Ca ICP reference solution (Ca ICP). Every sample was measured at least four times during a session and the mean value used for further calculations. The Ca isotopic composition is reported as δ^44/42^Ca in parts per thousand (‰):

(1) $$\delta^{44/42}\mathrm{Ca}\;\left(‰\right)=\left[{\left({}^{44}\mathrm{Ca}/{}^{42}\mathrm{Ca}\right)}_{\text{Sample}}/{\left({}^{44}\mathrm{Ca}/{}^{42}\mathrm{Ca}\right)}_{\text{Reference}}\right]–1$$

Further tests confirmed that there is negligible isotope fractionation during purification (<0.01‰). Although almost all Sr was removed from the samples during chemical preparation we monitored samples for doubly charged Sr (^84^Sr, ^86^Sr and ^88^Sr) that can interfere with the measurement of ^42^Ca, ^43^Ca and ^44^Ca, respectively (cf. (Morgan et al., 2011)). We applied several criteria to reject data of a single measurement, a single sample and even whole sequences. Following Morgan et al. (Morgan et al., 2011) a single measurement was rejected when |δ^44/42^Ca – 2·δ^43/42^Ca| > 0.2‰. A sample measurement (average of 4 to 5 single measurements) was rejected when the average intensity was outside a 70–130% intensity window when compared to the average intensity of the reference solution from the same batch. A complete sequence was rejected when more than one of the measured international reference materials deviated >0.2‰ from the literature value or the data did not fall along the mass-dependent fractionation line. The continuous measurement of international standard materials in the course of this study showed that they are in line with published values in the literature.

### 2.4 Statistical analysis

**Fig. 1:**
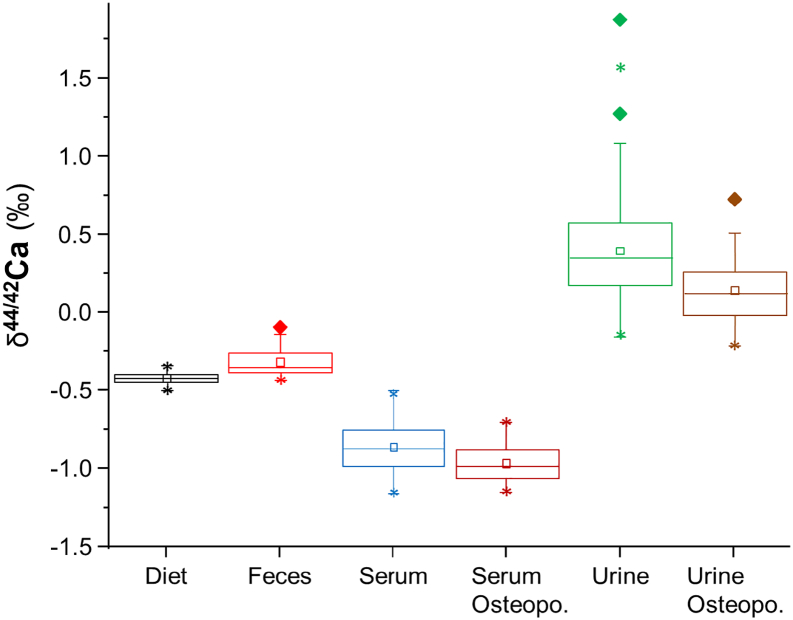
Ca isotope box plot values in women with (*n* = 14) and without osteoporosis (*n* = 66). Calcium isotope values in blood and serum are box plotted with their corresponding mean values diet, feces and calculated mean value for bone (see details in the text). There is no statistical difference in Ca isotope values between the two groups concerning the diet (*p* = 0.300) or the feces (*p* = 0.600). However, women suffering from DXA diagnosed osteoporosis showed significantly lower δ^44/42^Ca_Blood_ (*p* < 0.001) and δ^44/42^Ca_Urine_ (*p* = 0.004) values than those not suffering from osteoporosis. Boxes mark the 25% and 75% quartile, lines mark the median and squares mark the mean value. Diamonds mark outliers and stars mark the 1% and 99% limit of the data.

Quantitative values evaluated in the present diagnostic study were descriptively presented as mean and standard deviation (SD), minimum and maximum, as well as quartiles. Nominally scaled values were displayed in absolute and percent frequencies. Checking the distribution of quantitative study variables by Kolmogorov-Smirnov test, significant deviations from normality were considered. Differences between healthy controls and women with osteoporosis were evaluated using Mann-Whitney *U* test and were graphically shown in box-whisker plots (Fig. 1). Associations between different study variables were measured by nonparametric Spearman rank correlation coefficients (Table 1, Table 2).

**Table 1:** Comparison of women without and with osteoporosis.

Characteristics	Women without osteoporosis *n* = 66	Women with osteoporosis *n* = 14	*p*
Age	69.7 ± 5.4	69.9 ± 5.5	0.800
Total Ca (g/day)	0.99 ± 0.28	1.15 ± 0.39	0.140
Ca from milk (g/day)	0.42 ± 0.8	0.53 ± 0.20	0.005
[Ca]_Blood_ (mmol/l)	2.39 ± 0.10	2.42 ± 0.10	0.410
[Ca]_Urine_ (mmol/l)	1.96 ± 1.39	2.58 ± 1.26	0.042
[Ca]_Urine_/Creatinine ([mmol·dl]/[l·mg])	0.031 ± 0.04	0.048 ± 0.09	0.014
**δ^>44/42^Ca_Feces_(‰)**	−0.31 ± 0.09	−0.33 ± 0.11	0.598
DXA T-scores	−0.98 ± 0.9	−3.06 ± 0.8	–
δ^44/42^Ca_Blood_ (‰)	−0.84 ± 0.14	−0.99 ± 0.10	<0.001
δ^44/42^Ca_Urine_ (‰)	0.35 ± 0.33	0.10 ± 0.21	0.004
CRP (hs)	2.57 ± 1.95	1.65 ± 0.24	0.024
CTX (μg/l)	0.46 ± 0.17	0.57 ± 0.06	0.045
CTX/P1NP	0.009 ± 0.002	0.01 ± 0.002	0.020

Note: all reported uncertainties are 1 SD.

**Table 2:**
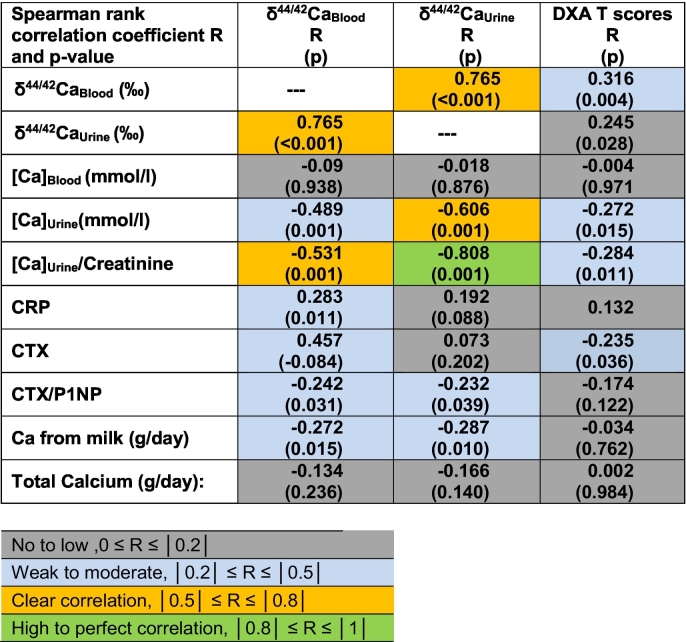
Pairwise Spearman rank correlations between parameters of mineral metabolism.

Note: Color coding emphasizes the degree of statistical significance of the correlations. The δ^44/42^Ca_Blood_- and δ^44/42^Ca_Urine_-values as well as DXA T-scores are compared (Spearman correlation) with parameters of mineral metabolism. Only parameters where at least one pair showed statistical significance are shown.

**Fig. 2:**
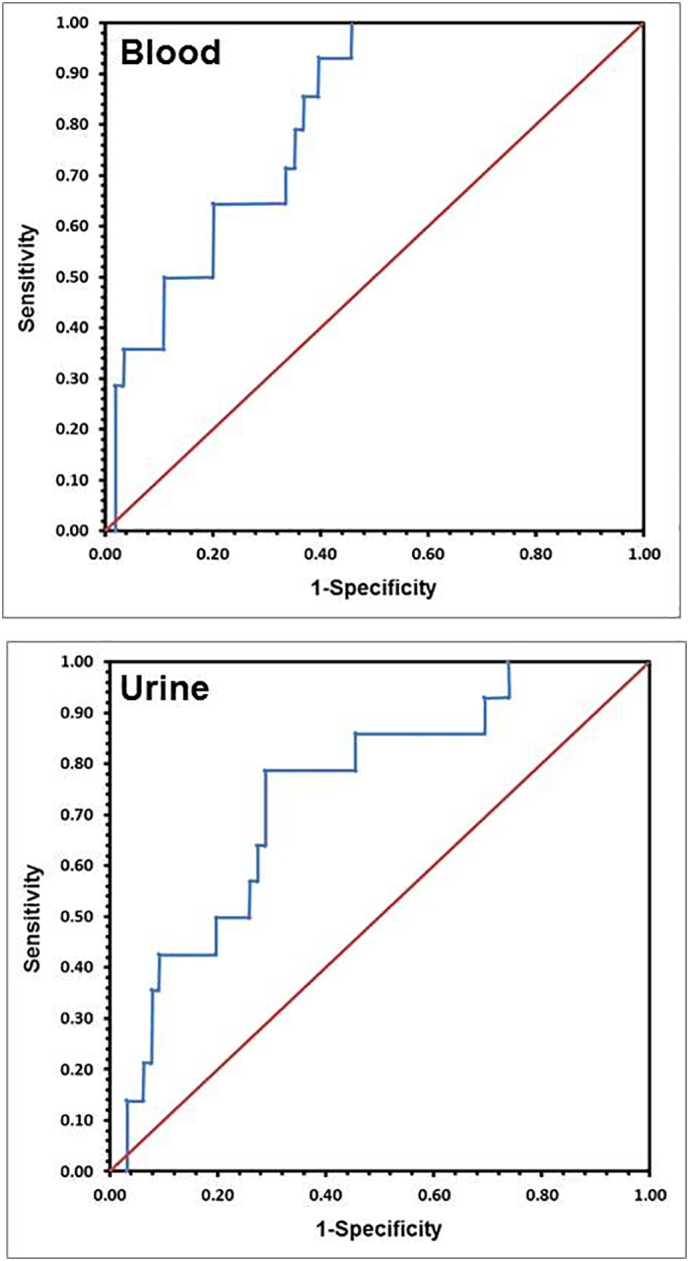
ROC curves for Ca isotope values in blood and urine for the prediction of osteoporosis. The best cut off in blood for δ^44/42^Ca_Blood_ values was found at about −0.85**‰** which predicts osteoporosis (without vitamin d deficiency) for values lower or equal with a sensitivity of 100% and a specificity of 54.5%.The best cut off value for Ca isotopes in urine for δ^44/42^Ca_Urine_ was found at about 0.16**‰** which predicts osteoporosis (without vitamin d deficiency) for values lower or equal to a sensitivity of 78.6% and specificity of 71.2%.

Cut-off values for Ca isotopes in blood and urine were determined by receiver operating characteristics (ROC) analyses (Fig. 2) using the Youden criterion. Diagnostic performance was measured as sensitivity, specificity and likelihood ratio referenced to the DXA T-score values as gold standard. Predictive values were evaluated in relation to the osteoporosis prevalence in this study. The central diagnostic meaning of Ca isotopes in combination with other influencing factors was assessed by a stepwise logistic regression analysis using backward elimination (Table 3a, Table 3b).

**Table 3a:** Criteria to assess the diagnostic power of Ca isotopes in blood (δ^44/42^Ca_Blood_).

Serum δ^44/42^Ca: Osteoporosis without Vitamin D deficiency	Osteoporosis without Vitamin D deficiency		Total
	No	Yes	
No (>−0,8475)	36 (45,0%)	0 (0%)	36 (45%)
Yes (≤−0,8475)	30 (37,5%)	14 (17,5%)	44 (55%)
Total	66 (82,5%)	14 (17,5%)	80 (100%)


Quality criteria	Value	95%-CI	
		Lower limit	Upper limit
Prevalence	17.50%	9.91%	27.62%
Sensitivity	100.00%	76.84%	100.00%
Specificity	54.55%	41.81%	66.86%
Positive predictive value	31.82%	18.61%	47.58%
Negative predictive value	100.00%	90.26%	100.00%
Positive likelihood ratio (LR+)	2.20	1.69	2.87
Negative Likelihood ratio (LR-)	0	0	0
Correct classification rate	62.50%	50.96%	73.08%
Youden-Index	54.55%	42.53%	66.56%

LR+ = Sensitivity/(1-Specificity); LR- = (1-Sensitivity)/Specificity.

**Table 3b:** Criteria to assess of diagnostic power of Ca isotopes in urine (δ^44/42^Ca_Urine_).

Urine δ^44/42^Ca: Osteoporosis without Vitamin D deficiency	Osteoporosis without Vitamin D deficiency		Total
	No	Yes	
No (>0,1638)	47 (58,8%)	3 (3,8%)	50 (62,5%)
Yes (≤−0, 1638)	19 (23,8%)	11 (13,8%)	30 (37,5%)
Total	66 (82,5%)	14 (17,5%)	80 (100%)


Quality criteria	Value	95%-CI	
		Lower limit	Upper limit
Prevalence	17.50%	9.91%	27.62%
Sensitivity	78.57%	49.20%	95.34%
Specificity	71.21%	58.75%	81.70%
Positive predictive value	36.67%	19.93%	56.14%
Negative predictive value	94.00%	83.45%	98.75%
Positive likelihood ratio (LR+)	2.73	1.71	4.36
Negative Likelihood ratio (LR-)	0.30	0.11	0.83
Correct classification rate	72.50%	61.38%	81.90%
Youden-Index	49.78%	25.67%	73.89%

LR+ = Sensitivity/(1-Specificity); LR- = (1-Sensitivity)/Specificity.

All tests were performed two sided with a significance level (p) of 5%. An alpha adjustment for multiple testing was not applied, and the results were interpreted accordingly. Statistical calculations were done using the statistical software IBM SPSS Statistics 25 (SPSS Inc. an IBM Company, Chicago, IL).

## 3 Results

### 3.1 Differences between the osteoporosis and healthy group

Of the 100 women screened, 20 had low vitamin D levels (≤25 nmol/l) and were excluded. The remaining 80 subjects were divided into 2 groups based on DXA scans (Table 1): 14 women with osteoporosis (≤−2.5 T-score) and 66 without osteoporosis (>−2.5 T-score) as controls. There was no difference in age between groups (*p* = 0.8). None of the women had fractures in the preceding five years. The Ca intake from milk was significantly different between groups, but total Ca was similar (*p* = 0.140_,_ Table 1). As expected, [Ca]_Blood_ were indistinguishable between groups (Table 1). There is also no significant isotope difference for the feces between the healthy and the osteoporosis group.

All clinical measures indicative of bone resorption and Ca loss (δ^44/42^Ca_Blood_, δ^44/42^Ca_Urine_, CRP, CTX, CTX/P1NP) and [Ca]_Urine_ were significantly different between the two groups (Table 1 and Fig. 1). In particular, Ca isotope values for blood (δ^44/42^Ca_Blood_) and urine (δ^44/42^Ca_Urine_) were significantly lower in women suffering from osteoporosis when compared to the control group (Fig. 1, Table 1).

The average DXA T-score value of the healthy and osteoporosis group are −0.98 ± 0.9 and −3.06 ± 0.8, respectively. For both lumbar spines and femoral neck average T score values are similar −0.7 ± 1.7 and − 0.7 ± 1.15, respectively. Lumbar spines and femoral neck values are also significantly correlated (*R* = 0.537, *p* < 0.0001, Pearson).

### 3.2 Correlations of Ca isotopes and DXA to markers of mineral metabolism and bone resorption

The Ca isotope values of blood (δ^44/42^Ca_Blood_) and urine (δ^44/42^Ca_Urine_) show a clear correlation (*r* = 0.765, *p* < 0.001, *n* = 80) to each other and a weak to moderate correlation to the DXA T-score values (see color coded values in Table 2). High to perfect as well as weak to moderate correlations also exist for the δ^44/42^Ca_Blood_ and δ^44/42^Ca_Urine_ values to [Ca]_Urine,_ [Ca]_Urine_/Creatinine, CRP, CTX, CTX/P1NP and Ca from milk (Table 2). The DXA values also show weak to moderate correlations to [Ca]_Urine,_ [Ca]_Urine_/Creatinine and to CTX.

### 3.3 ROC determined “cut-off values” for the Ca isotopes in blood and urine for women without vitamin D deficiency

To test the power of the δ^44/42^Ca_Blood_ and δ^44/42^Ca_Urine_ values in the diagnosis of osteoporosis statistically significant and optimized cut off values of about −0.85‰ and 0.16‰ were calculated from ROC analyses (Fig. 2, Table 3a, Table 3b). Women with δ^44/42^Ca_Blood_ and δ^44/42^Ca_Urine_ below their specific threshold values were considered to suffer from osteoporosis. Based on the cut-off values and relative to the DXA defined definition of osteoporosis the sensitivity to diagnose osteoporosis from the δ^44/42^Ca_Blood_ values was 100% whereas the specificity was calculated to be 54.5%. The sensitivity of the δ^44/42^Ca_Urine_ values to diagnose osteoporosis was 78.6% and specificity was 71.2%. Positive and negative predictive values as well as the related likelihood factors are reported in Table 3a, Table 3b.

In order to further test the predictive power of the Ca isotopes in blood and urine in differentiating women with osteoporosis versus those without osteoporosis, all measures with *p* < 0.05 from univariate analysis (Table 1) were entered into a stepwise logistic regression. In the first step all parameters associated with osteoporosis (δ^44/42^Ca_Blood_, δ^44/42^Ca_Urine_, CRP, XTX, CTX/P1NP) were entered into the statistical model. Stepwise exclusion of parameters improved the regression model and only δ^44/42^Ca_Blood_ remained as the most significant and independent predictor for osteoporosis (*p* < 0.001).

### 3.4 ROC determined “cut-off values” for the Ca isotope biomarker in blood and urine for the entire population

Repeating the statistical analysis for all women in this study and ignoring their vitamin D status (*n* = 100) showed that the best cut off value for δ^44/42^Ca_Blood_ values were −0.85‰ again which predicts osteoporosis with a sensitivity of 94% and a specificity of 54%. These values are very similar to the values calculated for women without vitamin deficiency. Similarly for urine the best cut off δ^44/42^Ca_Urine_ value was 0.23‰ which predicts an osteoporosis with a sensitivity of 72% and specificity of 67.1% again comparable to the values in women without Vitamin D deficiency.

## 4 Discussion

In accordance with earlier studies (Skulan et al., 2007; Skulan and DePaolo, 1999b; Morgan et al., 2012b) our results imply that in osteoporotic women a greater proportion of the Ca originates from bones which changed their δ^44/42^Ca_Blood_ and δ^44/42^Ca_Urine_ values without changing their corresponding [Ca]_Blood_ concentration but increasing their [Ca]_Urine_ and [Ca]_Urine_/Creatinine values (Table. 1). Latter observation indicates a higher loss of Ca from the bones in osteoporotic women when compared to the healthy control.

Statistical analyses show that the ROC optimized sensitivity is 100% corresponding to a negative predictive value (NPV) of ~100% for δ^44/42^Ca_Blood_. This indicates that the false-positive chance of still having osteoporosis although the δ^44/42^Ca_Blood_ values is above the ROC determined threshold value of −0.85‰ corresponds to zero and osteoporosis can be excluded. For δ^44/42^Ca_Urine_ a NPV of ~94% indicates that the false-positive chance of still having osteoporosis although the δ^44/42^Ca_Urine_ value is above the threshold value of 0.16 is still 6%. The corresponding negative likelihood factors are then 0 and 0.3 for δ^44/42^Ca_Blood_ and δ^44/42^Ca_Urine_, respectively, which qualifies them as good negative predictive biomarkers for the exclusion of osteoporosis.

However, as a consequence of the low specificity of ~55% the positive predictive value (PPV) is only about ~32% for δ^44/42^Ca_Blood_ and ~37% for δ^44/42^Ca_Urine_. This means that subjects having δ^44/42^Ca_Blood_ and δ^44/42^Ca_Urine_ below the corresponding ROC optimized threshold values still have a false-negative chance of ~30 to 40% of not having osteoporosis. This means the Ca isotope biomarker tends to predict more test persons to suffer from osteoporosis than actually determined by DXA.

**Fig. 3:**
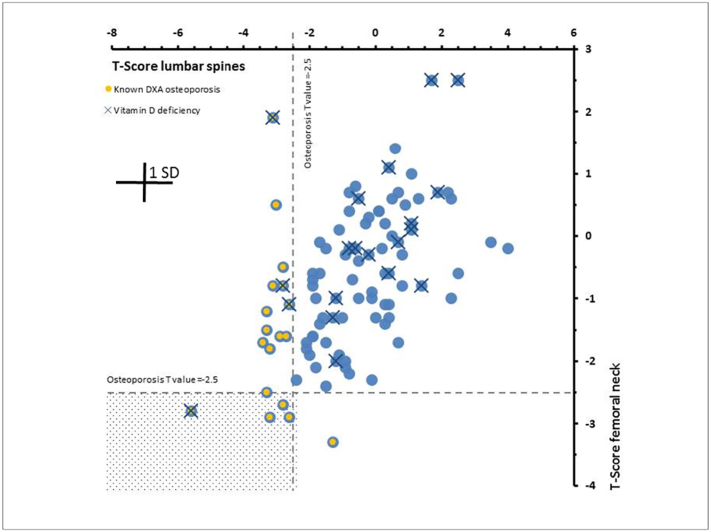
Correlations between DXA T-scores from femoral neck and lumbar spine for all 100 subjects. Broken horizontal and vertical lines represent T-score threshold values of −2.5 for the diagnosis of osteoporosis. DXA diagnosed osteoporosis and vitamin D deficiencies are marked. Osteoporosis was diagnosed in total of 18 cases. Only in 5 cases osteoporosis was diagnosed based on T-score values of both, femoral neck and lumbar spine (marked area). Only in one case osteoporosis is based on the femoral neck alone and in 12 cases lumbar spines alone indicated osteoporosis.

The apparent discrepancy in predicting osteoporosis between DXA as the gold standard and the Ca isotope biomarker may be reconciled taking the different methodological approaches into account. Osteoporosis induced bone Ca loss may not affect all bones simultaneously and to the same degree. In our study, BMD heterogeneity is demonstrated by the fact that of the 18 cases, neglecting vitamin D influence, of osteoporosis diagnosed by DXA, five were jointly based on the lumbar spine and the femoral neck, while 12 were based on lumbar spine DXA alone and one on the femoral neck DXA alone (Fig. 3). Obviously there is a distinct heterogeneity in the extent of bone demineralization from one bone to the other, with varying degrees of involvement in different bones and in different individuals. This is also supported by the observation that most bone fractures occur in women with normal or osteopenic BMD (Cranney et al., 2007) and there are a growing number of reports suggesting that many women who fracture have BMD higher than that usually associated with osteoporosis ((Wainwright et al., 2005) and references therein).

In contrast the Ca isotope biomarker reflects the current status and dynamics of the mineralization/demineralization process of the whole skeleton and not of a single bone. Values of δ^44/42^Ca_Blood_ and δ^44/42^Ca_Urine_ below the threshold value do not necessarily indicate an acute osteoporosis but rather a significant contribution of bone Ca to the blood as it is expected for osteoporosis and eventual net loss of Ca via the urine. Latter ongoing process may then eventually result in a DXA measurable osteoporosis in a later stage. Hence, the Ca isotopes measured in blood and urine reflects the net flux of bone Ca and the balance between bone mineralization and demineralization whereas DXA determines a distinct BMD status at a certain time. In this regard, the Ca isotope biomarker technique and DXA probably complement and extend each other.

Based on the statistical values of the determined values of sensitivity and specificity in our study into we propose that in future individuals who fall below the ROC determined Ca isotope biomarker threshold values in blood and urine still undergo DXA examination to determine their individual risk of bone fracture. However, based on its high sensitivity to exclude osteoporosis in individuals with δ^44/42^Ca_Blood_ and δ^44/42^Ca_Blood_ values above the threshold limit, DXA measurements and exposure to radiation may be avoided.

### 4.1 Quantitative model for calcium mineralization and demineralization in humans

**Fig. 4:**
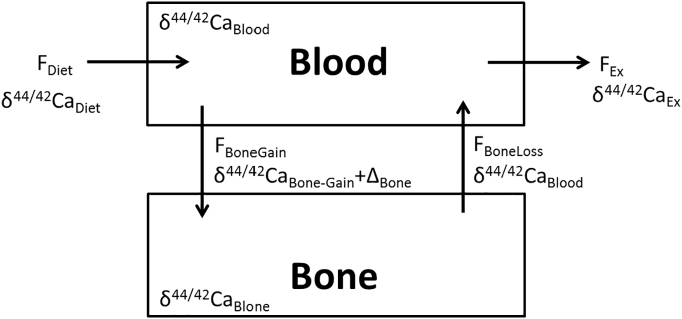
Simplified transport model for Ca isotopes between blood and bones in humans. In this model, there is no calcium isotopic fractionation associated with absorption, excretion of dietary calcium and dissolution of bones. Calcium incorporated into bone is derived from the blood, and there is a fractionation (Δ_Bone_) associated with the formation of bones.

**Fig. 5:**
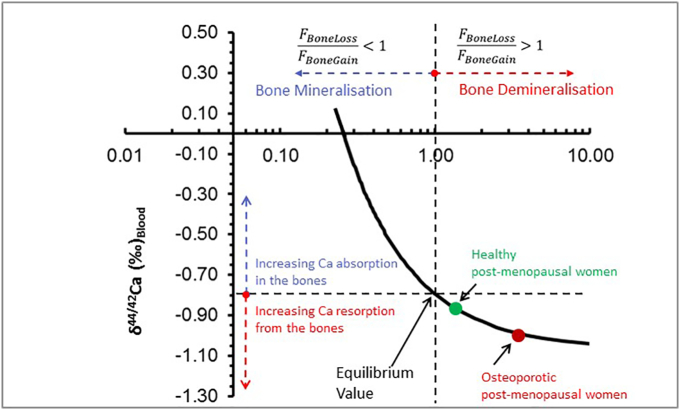
In this diagram the blood Ca isotope values are shown as a function of the F_Bone-Loss_/F_Bone-Gain_ ratios. An F_Bone-Loss_/F_Bone-Gain_ of 1 corresponds to the equilibrium value at which F_Bone-Loss_ equals F_Bone-Gain_. Above the equilibrium value Ca isotope values indicate a net gain of Ca from the blood and below 1 Ca isotopes indicate a net loss of Ca from the bones. The blue point marks the average Ca isotope composition of blood (δ^44/42^Ca_Blood_) for the healthy post-menopausal women (F_Bone-Loss_/_FBone-Gain_ ~1.3) and the red point marks the average composition of the osteoporotic post-menopausal women (F_Bone-Loss_/_FBone-Gain_ ~3.3). (For interpretation of the references to color in this figure legend, the reader is referred to the web version of this article.)

The results of our study show that the Ca isotopic composition of blood and urine strongly affected by mass-dependent Ca isotope fractionation associated with bone formation (Fig. 1). Bone is typically enriched in isotopically light Ca relative to the diet, blood and urine in terms of their Ca isotope ratios. With the following model, we demonstrate the quantitative relationship between Ca gain and loss in the bones and the measured δ^44/42^Ca_Blood_ and δ^44/42^Ca_Urine_ values (Fig. 4, Fig. 5). For simplicity in the model we focus on the δ^44/42^Ca_Blood_ value only. A similar model can also be designed for the δ^44/42^Ca_Urine_ values.

The simple two-compartment model for calcium use, transport, and fractionation in humans presented here is based on an earlier version by (Skulan and DePaolo, 1999b) on vertebrates and as well as by (Heuser and Eisenhauer, 2010) on humans. It is used to illustrate the quantitative relationship between skeletal Ca balance concerning bone mineralization and demineralization and the Ca isotope composition of blood (δ^44/42^Ca_Blood_) (Fig. 4, Fig. 5). The parameters in the model are (i) F_Diet_ and δ^44/42^Ca_Diet_, Ca flux and isotope composition of dietary calcium entering the body; (ii) F_Bone-Gain_ and δ^44/42^Ca_Bone-Gain_, Ca flux from the blood to the bone and its Ca isotope composition; (iii) F_Bone-Loss_ and δ^44/42^Ca_Bone-Loss_, Ca flux out of the bone and its isotope composition; (iv) F_ex_ and δ^44/42^Ca_ex_, Ca flux and isotope composition of the excreted Ca from the organism; and (v) the term Δ_Bone_ reflects the Ca isotope fractionation during Ca precipitation in the bone during mineralization. This conceptual model depicted in Fig. 4 results in a differential Eq. (1) which can be rearranged showing the dependency of δ^44/42^Ca_Blood_ from the Ca fluxes into (F_Bone-Gain_) and out of the bones (F_Bone-Loss_).

(2) $$\frac{\delta^{44/42}{\mathrm{Ca}}_{\mathrm{Blood}}}{\mathrm{dt}}=F_{\mathrm{Diet}}∙\left[\delta^{44/42}{\mathrm{Ca}}_{\mathrm{Diet}}-\delta^{44/42}{\mathrm{Ca}}_{\mathrm{Blood}}\right]\;+F_{\mathrm{Bone}-\mathrm{Loss}}∙\left[\delta^{44/42}{\mathrm{Ca}}_{\mathrm{Bone}-\mathrm{Loss}}-\delta^{44/42}{\mathrm{Ca}}_{\mathrm{Blood}}\right]\;-F_{\mathrm{Bone}-\mathrm{Gain}}∙{∆}_{\mathrm{Bone}}-F_{\mathrm{Ex}}∙\left[\delta^{44/42}{\mathrm{Ca}}_{\mathrm{Ex}}-\delta^{44/42}{\mathrm{Ca}}_{\mathrm{Blood}}\right]$$

In steady state ($\frac{\delta^{44/42}{\mathrm{Ca}}_{\mathrm{Blood}}}{\mathrm{dt}}=0$) and assuming that the term $F_{\mathrm{Ex}}∙\left[\delta^{\frac{44}{42}}{\mathrm{Ca}}_{\mathrm{Ex}}-\delta^{\frac{44}{42}}{\mathrm{Ca}}_{\mathrm{Blood}}\right]\;$ is actually close to zero Eq. (2) can be rearranged:

(3) $$\delta^{44/42}{\mathrm{Ca}}_{\mathrm{Blood}}=\frac{F_{\mathrm{Diet}∙}\delta^{44/42}{\mathrm{Ca}}_{\mathrm{Diet}}+F_{\mathrm{Bone}-\mathrm{Loss}}∙\delta^{44/42}{\mathrm{Ca}}_{\mathrm{Bone}-\mathrm{Loss}}-F_{\mathrm{Bone}-\mathrm{Gain}}∙{∆}_{\mathrm{Bone}}}{\left(F_{\mathrm{Diet}}+F_{\mathrm{Bone}-\mathrm{Loss}}\right)}$$

Define: $R=\frac{F_{\mathrm{Bone}-\mathrm{Gain}}}{F_{\mathrm{Bone}-\mathrm{Loss}}}$

Then, rearranging of 3:

(4) $$\delta^{44/42}{\mathrm{Ca}}_{\mathrm{Blood}}=\frac{F_{\mathrm{Diet}∙}\delta^{44/42}{\mathrm{Ca}}_{\mathrm{Diet}}+F_{\mathrm{Bone}-\mathrm{Loss}}∙\delta^{44/42}{\mathrm{Ca}}_{\mathrm{Bone}-\mathrm{Loss}}-F_{\mathrm{Bone}-\mathrm{Loss}}∙R∙{∆}_{\mathrm{Bone}}}{\left(F_{\mathrm{Diet}}+F_{\mathrm{Bone}-\mathrm{Loss}}\right)}$$

In Eq. (4) the values for δ^44/42^Ca_Diet_ (−0.41‰), δ^44/42^Ca_Bone_ (−1.14‰) and Δ_Bone_ (−0.3‰) are already known from our study data (Fig. 1), tables and the discussions above. Furthermore, values for the absorbed amount of diet (F_Diet_) are taken to be 0.15 g_Ca_/day as calculated from our data and in accordance with literature values (Heaney, 2006). The two unknowns are the Ca fluxes from the blood to the bones (F_Bone-Gain_) and back from the bone to the blood (F_Bone-Loss_). However, F_Bone-Gain_ can be expressed as a function of F_Bone-loss_ (Eq. (4)) which allows us to present the δ^44/42^Ca_Blood_ values as a function of the F_Bone-Loss_ to F_Bone-Gain_ ratio (F_Bone-Loss_/F_Bone-Gain_) arbitrarily assuming that the daily loss of F_Bone-Loss_ is about 1.5 g_Ca_/day (Bushinsky, 2010). Then Eq. (3) can unambiguously solved as a function of the F_Bone-Loss_/F_Bone-Gain_ ratio.

From Fig. 5 it can be seen that the δ^44/42^Ca_Blood_ values decrease as a function of increasing F_Bone-Loss_/F_Bone-Gain_ values reflecting the increasing contribution of isotopically light Ca from the demineralizing bones to the blood (F_Bone-Loss_/F_Bone-Gain_ ratio > 1). In contrast increasing Ca fluxes from the blood to the bone (F_Bone-Loss_/F_Bone-Gain_ ratio > 1) correspond to increasing the δ^44/42^Ca_Blood_ values. Equilibrium of input and output Ca fluxes (F_Bone-Loss_ = F_Bone-Gain_) in the blood are reflected by an F_Bone-Loss_/F_Bone-Gain_ value of 1 (Fig. 5).

From our study and model values a δ^44/42^Ca_'Blood_ equilibrium value of about −0.80‰ can be calculated which is closely corresponding to the average δ^44/42^Ca_'Blood_ value of the healthy post-menopausal women of −0.84 ± 0.14‰ (Table 1). The corresponding calculated F_Bone-Loss_/F_Bone-Gain_ ratio of ~1.3 (Fig. 5) indicates that these women loose about 30% more Ca by bone demineralization than they gain by mineralization. The average δ^44/42^Ca_'Blood_ value of −0.99 ± 0.10‰ (Table 1) of the osteoporotic post-menopausal women corresponds to a value F_Bone-Loss_/F_Bone-Gain_ ratio of ~3.3 (Fig. 5) indicating that this group of women losses about 330% more Ca than it gains by bone mineralization. Note, that these losses just reflect the exchange of Ca between bones and blood and are not taking the recycling of Ca in the kidney into account.

Based on this simple quantitative model, it is made clear that the δ^44/40^Ca_Blood_ value in humans will provide a quantitative indication of the relative rates of mineral formation (F_Bone-Gain_) and mineral dissolution (F_Bone-Loss_). Organisms experiencing F_Bone-Loss_/F_Bone-Gain_ value of >1 reflecting bone demineralization should have δ^44/40^Ca_Blood_ values that are below the equilibrium value, whereas organisms experiencing F_Bone-Loss_/F_Bone-Gain_ values <1 reflecting mineralization are characterized by δ^44/40^Ca_Blood_ value values above the equilibrium value.

## 5 Limitations of our study

• We were unable to achieve the target number of women with osteoporosis as estimated from initial assumptions.
• A study examining a larger population of women, and ideally with repeated measurements of Ca isotope fractionation in a longitudinal follow-up study design would be recommended.
• Our study does not examine the association of Ca isotope measurements against fracture outcomes, and this would be an important aspect for future studies.
• Our study population was also limited to post-menopausal women only, and future studies must include other groups too.
• Other factors may potentially influence the Ca isotope composition, such as changes in the amount or type of diet, changes in the rates of renal Ca reabsorption as expected in renal impairment and the rates of Ca turnover require further investigation.
• The effects of anti-resorptive therapy such as bisphosphonates or denosuamb on changes in BMD will also be the subject of future studies.

## 6 Summary of the results and discussion

• Bone resorption that leads to a net loss of bone Ca in women with osteoporosis cause a decrease in the Ca isotope values of blood and urine.
• Ca isotope ratios measured in blood (δ^44/42^Ca_Blood_) and urine (δ^44/42^Ca_Urine_) reflects the Ca balance of the whole skeleton rather than of individual bones.
• Ca isotopes in blood and urine correlated highly significantly with each other and with the corresponding DXA measurements. Diagnostic sensitivity for the δ^44/42^Ca_Blood_ values was 100% and for the δ^44/42^Ca_Urine_ values ~72%.
• ROC optimized cut-off values (δ^44/42^Ca_Blood_ <~−0.85, δ^44/42^Ca_Urine_ <~0.16) significantly discriminated individuals with and without osteoporosis.
• Logistic regression analysis showed that the Ca isotope composition of blood (δ^44/42^Ca_Blood_) was the strongest and independent biomarker for osteoporosis and the addition of other clinical parameters did not improve its diagnostic power.
• The apparently low specificity of ~55% of Ca isotopes in blood to predict osteoporosis on DXA is inherent to the methodological approaches in measuring bone mineralization by DXA and the Ca isotope biomarker.

## Transparency document

Transparency document. Image 1
